# Supplementary material for: Reproductive Biology and Breeding Systems of Two Opisthopappus Endemic and Endangered Species on the Taihang Mountains
Source: Plants (Basel). 2023 May 11;12(10):1954. doi: 10.3390/plants12101954 (PMC10222883; doi:10.3390/plants12101954)
Supplement: Supplementary file 1 [file plants-12-01954-s001.zip › plants-2252724-supplementary.pdf]

plants

MDPI

Supplementary Materials

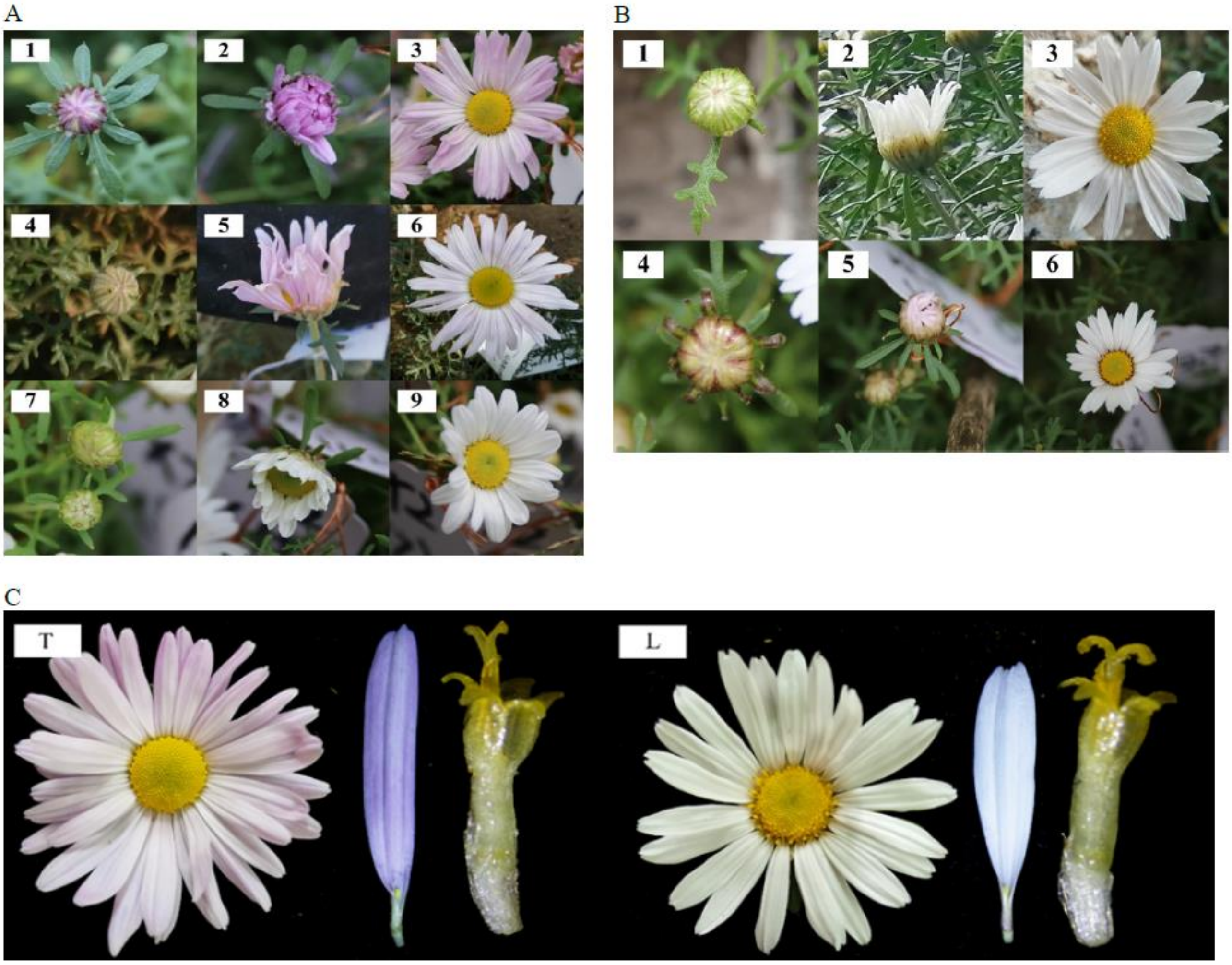

Figure S1. Floral traits of *Opisthopappus taihangensis* and *Opisthopappus longilobus*. (A): Different colors of *O. taihangensis* undeveloped capitulum buds (1–3 purple bud, purple ligules, light purple capitulum; 4–6 green bud, pink ligules, light pink capitulum; 7–9 green bud, white ligules, white capitulum); (B): Different colors of *O. longilobus* undeveloped capitulum buds (1–3 green bud, white ligules, white capitulum; 4–6 purple bud, light pink ligules, white capitulum), (C): T: *O. taihangensis* capitulum, ligule (1.5x), floret (4x); L: *O. longilobus* capitulum, ligule (1.5x), floret (4x)

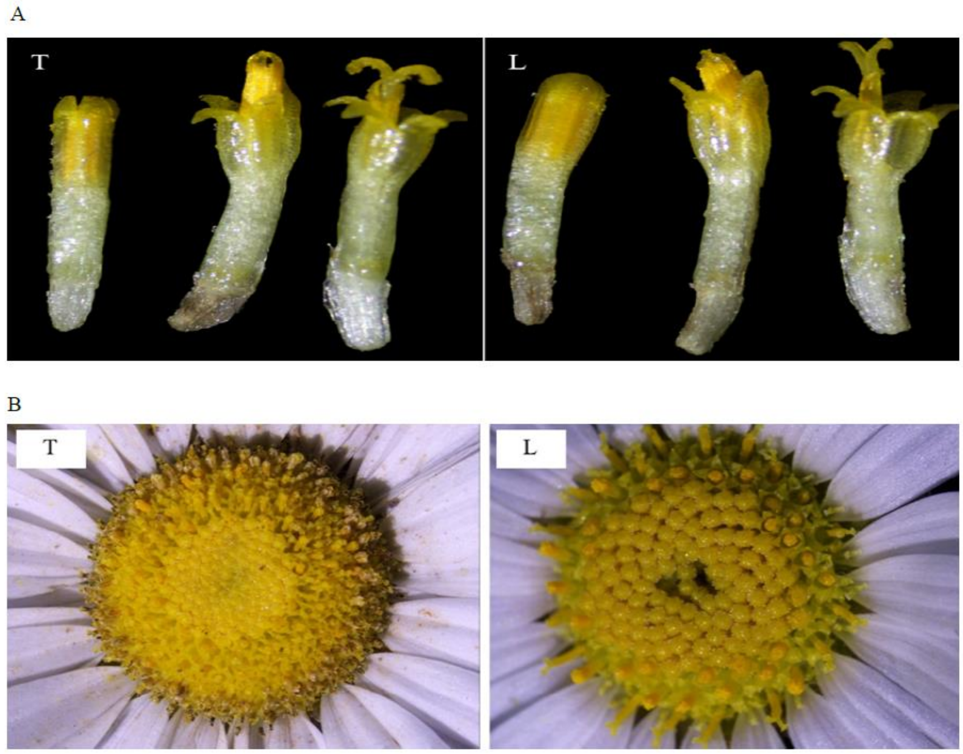

Figure S2. Dynamic changes of florets of *Opisthopappus taihangensis* and *Opisthopappus longilobus* flower maturation (T: *O. taihangensis*; L: *O. longilobus*). (A): Changes in florets; (B): unfolding of florets.

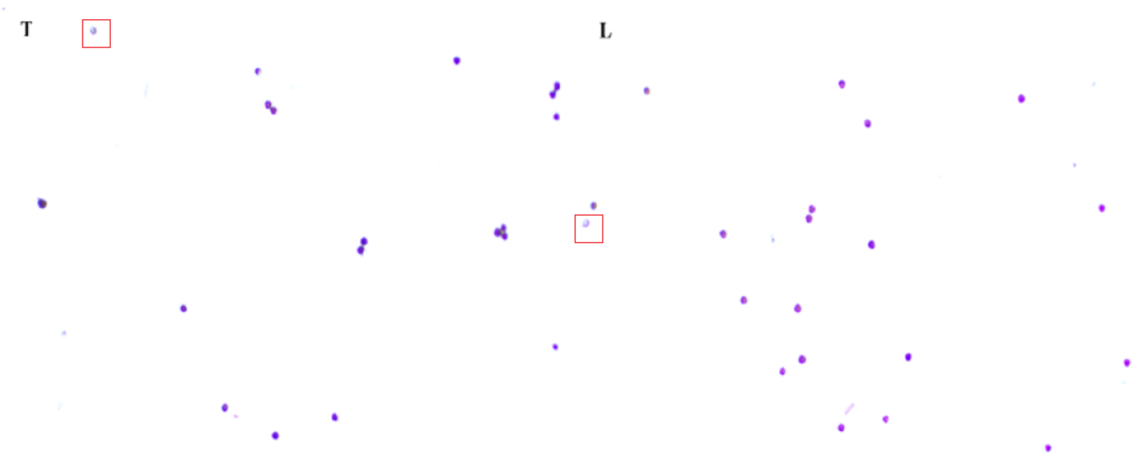

Figure S3. Pollen vitality of *Opisthopappus taihangensis* and *Opisthopappus longilobus* (5x) (T: *O. taihangensis*; L: *O. longilobus*; Red box: weak or inactive pollen vitality).

Table S1. Daily activity of visiting insects.

	10：00-11：00	11：00-12：00	12：00-13：00	13：00-14：00	14：00-15：00	15：00-16：00	16：00-17：00	17：00-18：00
O. taihangensis	3.05±0.05 a	3.04±0.04 a	12.05±0.38 a	27.10±0.05 a	15.05±0.05 a	15.02±0.05 a	6.15±0.05 a	1.13±0.03 a
O. longilobus	3.00±0.04 a	1.04±0.37 b	9.00±0.01 b	21.04±0.08 b	3.14±0.06 b	3.13±0.05 b	1.00±0.04 b	1.04±003 a

Note: a, no-significant variation; b, significant variation; $p < 0.05$.

Table S2. Visit flower frequency of insects at different sites.

Orders	Family	Species	LFT Visiting frequency（times/h）	LFL Visiting frequency（times/h）	SNST Visiting frequency（times/h）	SNSL Visiting frequency（times/h）	XTST Visiting frequency（times/h）	XTSL Visiting frequency (times/h）
Diptera	Syrphidae	*Episyrphus balteatus*	-	-	9.05±0.03	4.09±0.05	18.07±0.06	12.12±0.06
		Ischiodon scutellaris Fabricius	5.03±0.04	3.13±0.04	-	-	11.05±0.05	9.09±0.06
		Eristalis tenax	-	-	-	-	34.06±0.06	-
		Eristalis arvorum	-	-	-	-	17.14±0.06	-
	Cordyluridae	*Scathophaga stercoraria*	-	-	2.02±0.03	-	-	3.06±0.03
Hymenoptera	Vespidae	*Vespula vulgaris*	9.12±0.05	9.09±0.04	-	-	18.13±0.03	-
		Vespula flaviceps	-	-	-	-	11.12±0.03	5.12±0.05
		Vespa velutina	-	-	3.07±0.05	-	-	-
		Parasitoid wasp	-	-	4.03±0.05	-	-	-
Coleoptera	Cetoniidae	*Oxycetonia jucunda* Faldermann	-	-	2.13±0.06	1.08±0.03	-	-
Lepidoptera	Lycaenidae	*Aricia mandschurica Staudinger*	-	-	-	-	-	0.68±0.04

Lepidoptera	Nymphalidae	*Polygonia caureum*	-	-	-	0.85±0.03	-	-
Rhynchota	Pentatomidae stinkbug	*Halyomor-pha halys*	-	-	0.98±0.03	-	-	-
